# Genetic polymorphisms in *HMGCS1* gene and its association with slaughter characteristics, meat quality, and organ coefficients in Guizhou white goats

**DOI:** 10.5713/ab.24.0499

**Published:** 2025-01-16

**Authors:** Bo Zhou, Jiaqi Chen, Ziyang Li, Huan Liu, Jiali Xu, Houqiang Xu, Yong Ruan

**Affiliations:** 1 Key Laboratory of Animal Genetics, Breeding and Reproduction in the Plateau Mountainous Region, Ministry of Education, Guizhou University, Guiyang, China; 2 Guizhou Provincial Key Laboratory of Animal Genetics, Breeding and Reproduction, Guizhou University, Guiyang, China; 3 College of Animal Science, Guizhou University, Guiyang, China

**Keywords:** Genetic Polymorphism, Guizhou White Goats, *HMGCS1* Gene, Meat Quality, Organ Coefficients, Slaughter Characteristics

## Abstract

**Objective:**

This study aimed to identify polymorphisms in the gene encoding the 3-hydroxy-3-methylglutaryl-CoA synthase 1, *HMGCS1*, and analyze their association with slaughter characteristics, meat quality, and organ coefficients in Guizhou white goats.

**Methods:**

A total of 153 twelve-month-old Guizhou white goats (78 male and 75 female) were included in the study. Slaughter characteristics, meat quality, and organ coefficients were assessed. Association analyses between genotypes and phenotypic traits were conducted using a generalized linear model.

**Results:**

Four polymorphic loci were identified, i.e., g.15523T>C, g.15530G>C, g.18413T>C, g.19711G>A in exons 5, 8, and 9 of the *HMGCS1* gene. Across all polymorphic loci, males of the same genotype generally exhibited significantly better slaughter traits compared to females of the same genotype (p<0.05). At the g.18413T>C locus, differences in shear force were observed between males and females of the same genotype and within the same sex across different genotypes (p<0.05). Organ coefficients were significantly higher in males of the same genotype compared to females (p<0.05). The g.15523T>C and g.15530G>C loci were found in strong linkage disequilibrium and significantly associated with intramuscular fat content (p<0.05). Fat content in diploid Hap2/2 individuals was significantly higher than in Hap1/1 and Hap1/2 (p<0.05). At the g.19711G>A locus, female goats with the CC genotype showed significantly higher levels of dry matter compared to male goats of the same genotype and female goats of other genotypes within the group. Organ coefficients for the liver and hooves in male goats were significantly higher than in females of the same genotype (p<0.05).

**Conclusion:**

The strongly linked loci g.15523T>C and g.15530G>C were significantly associated with intramuscular fat content and could be used as molecular markers for enhancing this trait in goat breeding programs, fostering the development of goat production.

## INTRODUCTION

Goat breeding has seen a growing emphasis on optimizing local breeds to meet the escalating demand for high-quality meat. Among them, the Guizhou white goat, with a history exceeding 2,000 years, is the oldest and most renowned meat goat breed in Guizhou Province. Recognized in the National Livestock and Poultry Breed Annals in 1993, this breed is primarily raised in Sinan, Yanhe and Wuchuan counties, located in the middle and lower reaches of the Wujiang River Basin in Guizhou Province [1]. The breed is characterized by high reproductive capacity, superior meat quality, mild mutton flavor, and excellent hide quality, with a population estimated at one million. In China, efforts are made to improve genetic diversities of Guizhou white goats. A conservation and breeding farm was established in 2007, combining core farm and scattered farmers’ efforts. Yanhe County had a 1,000-ewe conservation farm. Guizhou white goats were included in breed records in 1989, 1993, and 2011. They are an important germplasm resource for developing animal husbandry in Guizhou province [2]. Despite its popularity among consumers nationwide, this breed faces challenges such as slow growth rate and low feed conversion efficiency [3]. Consequently, it is essential to determine the slaughter performance and meat quality traits of Guizhou white goats and develop efficient breeding strategies for this breed [4].

Modern biotechnology and breeding techniques offer opportunities to improve the desirable characteristics of Guizhou white goats. Combining molecular breeding with traditional phenotypic selection is widely practiced in animal breeding, offering substantial practical value [5,6]. For instance, the g.133513422 C>T polymorphism in the *GPAT1* gene has been shown to significantly influence intramuscular fat content in the longissimus lumborum of pigs, suggesting that this single nucleotide polymorphism (SNP) might be considered a genetic marker for improving pork quality [7].

Slaughter characteristics, meat quality, and organ coefficients are crucial factors directly impacting livestock farming profitability. These low-heritability traits are influenced by genetics, environment, management, and nutrition [8,9].Guizhou white goats has exhibit unique traits, including large body size, high carcass weight, favorable slaughter percentages, rapid fattening rate, the ability to reach the appropriate slaughter weight in a shorter time than other breeds, and competitive market performance. Meat quality, delicious and delicate, good tenderness, light odor, rich in protein and other nutrients, in line with the pursuit of healthy food. The organ coefficient reflects the physiological state and health status of Guizhou white goats. The organs of Guizhou white goats develop well, and the important organs are matched with body weight, with good physiological function, which provides a guarantee for their healthy growth and good performance.

SNP analysis of genes and morphological traits offers a more precise and effective approach for breeding high-quality breeds within high fecundity populations, a SNP refers to a single nucleotide variation at a specific position in the genome that is present in a significant proportion of the population (usually>1%). It is the most common type of genetic variation among individuals and can occur within coding regions (exons), non - coding regions (introns), or regulatory regions of genes [10,11]. The *HMGCS1* gene, belonging to the HMGCS family, encodes a crucial enzyme located in the cytoplasm, which plays a vital role in cholesterol and ketone biosynthesis [12]. While the cytoplasmic *HMGCS1* regulates cholesterol biosynthesis [13,14], the mitochondrial HMGCS2 controls ketone body biosynthesis. Research on the *HMGCS1* gene has primarily focused on its role in the development of important diseases, including tumors, cardiovascular disease, and metabolic diseases, e.g., diabetes [15,16]. In livestock, *HMGCS1* has been associated with porcine skeletal muscle growth and development, suggesting it might be a potential candidate for the selection of improved production traits in pork [17]. In a previous study, it was found that the *HMGCS1* gene in adipose tissue might be involved in determining meat quality, suggesting it might play a role in the PPAR signaling pathway, which is required for energy production and adipogenesis [18]. Moreover, it has been shown that *HMGCS1* promotes male differentiation in chicken embryos by regulating cholesterol production [19]. In addition, studies on goats have demonstrated that the *HMGCS1* gene plays a crucial role in the synthesis of fatty acids and unsaturated fatty acids. Furthermore, stratification genes, including *CYP2C89*, *HMGCS2*, and *SULT1B1*, have been identified as potentially contributing to the production of goat mutton odor and flavor [20], hence suggesting that *HMGCS1* might be closely associated with important productive traits. However, the effect of polymorphisms in the *HMGCS1* gene on slaughter performance and meat quality traits in goats remains largely unexplored.

Therefore, the aim of this study was to evaluate slaughter characteristics, meat quality, and organ coefficients of Guizhou white goats, and explore the association between polymorphisms in the *HMGCS1* gene and these traits. The findings discussed herein provide guidance for improving slaughter characteristics and meat quality in goats using molecular breeding.

## MATERIALS AND METHODS

### Ethical approval

This study was approved by the Guizhou University Subcommittee of Experimental Animal Ethics (Approval no: EAE-GZU-2023-E045; EAE-GZU-2024-E052) and was conducted in compliance with all relevant China legislations.

### Animals and management

The experiments included 153 twelve-month-old healthy Guizhou white goats, obtained from Wuchuan Hongmu Goat Industry Co., Ltd., located in the Guizhou Province, China. The animal cohort comprised 78 male and 75 female goats. All animals were housed under similar conditions and strict environmental control, being fed a balanced diet comprising whole corn plant silage with a 50:50 concentrate-to-fodder ratio as detailed in Table 1. Feeding occurred at 08:00 and 17:00 daily. After a 15-day pre-feeding period, the feeding experiment lasted 90 days. Water was provided ad libitum, and all other management practices were carried out in accordance with the farm’s established protocols. Environmental conditions were maintained at 22°C with 50% humidity, complemented by adequate natural light and ventilation to simulate a natural habitat and minimize stress. Routine handling was conducted by trained personnel using low-stress techniques, and the facility was designed to minimize noise and disturbances. These practices supported the well-being and consistent behavior in goats, as well as ensured minimal environmental impact on growth and meat quality, therefore maximizing reliable and reproducible experimental results.

### Determination of slaughter characteristics

Animals were desensitized using electric shocks and subsequently slaughtered by exsanguination by severing the jugular vein and carotid artery to ensure complete blood drainage. This method was designed to ensure a quick and humane slaughter while minimizing stress and suffering to the animal. All animals underwent fasting for 24 h and water abstention for 2 h prior to weighing to determine pre-slaughter live weight. Carcass weight was measured post-slaughter and excluded the skin, head, carpal bones of the forelimbs, sections below the tarsal joint of the hind limbs, and internal organs, except for the kidneys and renal fat [21]. Net meat weight was defined as the combined weight of the muscle and fat on the carcass, including the kidneys and renal fat [22]. The loin muscle was extracted by making a transverse cut at the posterior edge of the 12th rib of the left half-carcass. The loin eye muscle area was calculated using the formula:


Loin eye muscle area=Longissimus muscle height×longissimus muscle width×0.7[
23].

The grade ruling value (GR value) refers to the tissue thickness at 11 cm from the midline of the back between the 12th and 13th ribs. The thickness of back fat refers to the fat thickness directly above the lumbar muscles between the 12th and 13th ribs. All measurements of the loin muscle height, width, GR value, and the thickness of back fat were conducted using digital calipers (Deli, Hangzhou, China).

Slaughtering percentage, a critical indicator of growth and slaughter performance, was calculated as:


Slaughtering percentage=Carcass weight/Pre-slaughter live weight×100[
24]

Net meat percentage and carcass net meat percentage, used to assess meat production capacity and slaughter quality, were determined using the formulas below:


$$\begin{array}{l} \text{Net meat percentage}=\text{Net meat weight}/\text{Pre-slaughter live weight}\times 100\\ \text{Carcass net meat percentage}=\text{Net meat weight}/\text{Carcass weight}\times 100 \end{array}$$[
25]

### Determination of meat quality

Meat quality parameters were evaluated in longissimus dorsi muscle and included the following: meat color, pH value (45 min), dry matter, protein content, fat content, drip loss rate, cooked meat rate, and shear force.

The *longissimus thoracis* muscle was excised between the 11th and 13th ribs immediately after slaughter to evaluate meat quality traits. Meat color parameters, including lightness (L*), red-green color (a*), and yellow-blue color (b*), were determined in the MiniScan EZ 4000 chroma meter (HunterLab, Reston, VA, USA). Meat pH was measured 45 min post-mortem using the PH100P pH meter (Harveson, Suzhou, China).

Subsequently, 100 g of meat was boiled in water for 30 min, cooled at room temperature for 20 min, and then weighed. The cooked meat rate was calculated as follows:


Cooked meat rate=Meat weight before cooked-Meat weight after cooked/Meat weight before cooked×100%

Then, fragments of the meat sample measuring 5 cm×3 cm×2 cm were placed in a water bath at 80°C until its center temperature [26–30] reached 70°C. The sample was removed and cut perpendicularly to the muscle fibers into fragments measuring 1.5 cm×1 cm×1 cm, and shear force was measured in a C-LM3 muscle tenderness meter (G&R Teck, Thousand Oaks, CA, USA) based on the agricultural industry standard of the People’s Republic of China.

A portion of 5 to 10 g of meat sample was obtained. Multiple samples were taken from the same batch of meat. Each sample was minced separately. For the determination of dry matter content, a subset of these samples was dried using the direct drying method. For measuring crude protein content, a different subset of samples was analyzed using the Kjeldahl method [31]. Similarly, for determining crude fat content, yet another subset of samples was processed using the Soxhlet extraction method [32]. This approach was taken to ensure that the results are representative and to minimize the risk of errors due to potential issues with a single sample. Drip loss rate was determined by suspending a 5 cm×3 cm×2 cm fragment of the longissimus thoracis sample (in a sealed plastic bag at 4°C for 24 h [33]. All methods employed herein were in accordance with the Chinese standard NY/T 1236-2006 (Specifications for Measuring Performance in Sheep and Goat).

### Determination of organ size

Various organs, including the liver, heart, lung, spleen, kidney, small intestine, large intestine, stomach, head and hoof, were weighed, and coefficients were calculated according to the formula: organ coefficient = organ weight/live weight before slaughter.

### DNA isolation

Blood samples were collected from the jugular vein of each animal included in the study prior to slaughter into EDTA tubes and stored at −20°C. DNA extraction from whole blood was performed using the E.Z.N.A. Blood DNA Kit (OMEGA Bio-Tek, Norcross, GA, USA) according to the manufacturer’s instructions. DNA quantity and quality were assessed using a spectrophotometer (Thermo Scientific, Waltham, MA, USA), and DNA samples were then stored at −20°C until further use.

### Polymerase chain reaction amplification and DNA sequencing analysis

Primers were designed for all exons of the *HMGCS1* gene of Guizhou white goats based on the sequence available in the GenBank database (accession number: NC_030827.1) using Primer Premier 5.0 software (PREMIER Biosoft International, Palo Alto, CA, USA). Designed primers were synthesized by Beijing, Genomics Technology Co., Ltd., China. Primer sequences, product sizes, and annealing temperatures are shown in Table 2.

The polymerase chain reaction (PCR) system (30 μL final volume) consisted of the following: 15 μL of 2×Taq PCR Star Mix (Yeasen Biotechnology, Shanghai, China); 1 μL of forward and reverse primers; 1 μL of DNA template; and 12 μL of ddH_2_O. The PCR program was as follows: pre-denaturation at 94°C for 3 min; denaturation at 94°C for 30 s, annealing at 60°C for 30 s, and extension at 72°C for 2 min, for a total of 35 cycles; followed by a final extension at 72°C for 7 min; and storage at 4°C. PCR products were analyzed after horizontal electrophoresis in 1.0% agarose gels and sent to Tsingke Biotechnology Co., Ltd., China, for sequencing, which was performed using Sanger method, the gold standard for SNP detection. DNAStar software (DNAStar Inc, Madison, WI, USA) was employed to compare sequencing results with NCBI original sequences to identify potential SNP loci.

### Statistical analysis

The presence of SNPs in the *HMGCS1* nucleotide sequence was identified by peak plotting of PCR sequencing reads using SeqMan software [34]. Wild-type and mutant sequences were aligned and analyzed using MegAlign and ClustalW software in the DNA Star package. Genotype and allele frequencies at each mutation locus were calculated directly, while Hardy-Weinberg equilibrium was assessed using the chi-squared (χ^2^) test. Polymorphism parameters, including homozygosity (*Ho*), heterozygosity (*He)*, the number of effective alleles (*Ne*), and polymorphic information content (*PIC*), were calculated as previously described elsewhere [35]. Linkage disequilibrium (LD) and haplotype analyses among SNPs were performed using the SHEsis Main11 software [36]. The degree of chain imbalance was evaluated using the r^2^ value, where r^2^ > 0.33 indicated a strong chain imbalance state [37]. Diplotype analyses were conducted based on identified haplotypes.

All experimental data were analyzed in SPSS v.25.0 (IBM, Armonk, NY, USA). Differences in slaughter and meat quality traits between male and female goats were assessed, and the associations between polymorphisms in the *HMGCS1* gene and slaughter characteristics, organ coefficients, and meat quality traits were analyzed.

Before statistical analysis, normality and homoscedasticity assumptions were tested. The Shapiro-Wilk test showed that the data approximately met the assumption of normality. Similarly, the Levene test indicates approximately equal variances. The generalized linear model is more flexible and can handle biased data, is applicable to multiple variables with different distributions, and the model handles outliers and heteroscedasticity better even when it is approximately normal. Therefore, we chose the generalized linear model for the analysis.

We first constructed the full model, which included all variables that could be associated with *HMGCS1* gene polymorphisms, sex, and other potential influencing factors such as environmental factors, management factors, and individual physiological characteristics. The full model provides a framework for comprehensive assessment of the overall impact of each factor to avoid missing important information. However, there are many dependent variables in the full model, which may have multicollinearity problems, and some variables have no significant influence on the dependent variables, which will make the model too complex and reduce the stability and interpretation. Therefore, we used stepwise regression to construct reduced models. All variables were first included in the model for preliminary fit, and the partial regression sum of squares was calculated for each variable. At each step, variables that contributed the most to the dependent variable and were statistically significant, such as p<0.05, were selected to enter the model, while variables already in the model were reevaluated. If a variable was no longer significant or its addition degraded the overall performance of the model (such as increasing the mean square error, AIC or BIC value, etc.), it was excluded. By continuously adding and removing variables, the model was gradually optimized to obtain a simplified model containing the most significant variables. While retaining key information, the complexity was reduced, and the stability and interpretability were improved, so that the main influencing factors and their mechanisms could be more clearly identified.

The analysis of gender and genotype was stratified. Firstly, a preliminary analysis was conducted in the overall sample to evaluate the overall influence trend of genotype, sex and interaction terms on each studied trait, and to understand the approximate role of gene polymorphism and sex factors at the population level. Next, for the male goat population, data were extracted separately, and only the influence of genotype variables on each trait was considered. Through fitting models and statistical tests (ANOVA), which genotypes in the male population were significantly associated with specific traits were determined. The above procedure was repeated for the female goat population to study the effect of genotype on each trait in females separately. Finally, the differences in the performance of the same genotype between male and female goats were compared, and the statistical test (two independent sample t test) was used to evaluate whether the trait difference was significant and explore whether it was related to gender-related physiological mechanisms. This hierarchical approach allows a more detailed analysis of the effects of sex and genotype and provides a comprehensive perspective on the gene-phenotype relationship.

All results are presented as means ± standard deviation. The generalized linear model used was as follows:


$$g(E(Y_{ijk}))=\beta_{0}+\beta_{1}G_{1i}+\beta_{2}G_{2i}+\beta_{3}S_{1j}+\beta_{4}G_{1i}S_{1j}+\beta_{5}G_{2i}S_{1j}+e_{ijk}$$

Where g(·) represents the link function, *E(Y_ijk_*) is the expectation of the dependent variable, *β_0_* is the intercept term, representing the expected value of the dependent variable when all independent variables are zero, *β_1_*, and *β_2_* represent the effects of genotypes *G_1_* and *G_2_* on the dependent variable, respectively, relative to the reference genotype *G_3_*. The determination of these genotype effects was based on comparing the means of the dependent variable across different genotypes. *β_3_* represents the effect of gender on the dependent variable. This was assessed by comparing the means of the dependent variable between male and female goats. *β_4_* and *β_5_* represent the effects of the interaction between genotypes *G_1_*, *G_2_*, and gender on the dependent variable. These interaction effects were evaluated to understand how the relationship between genotype and the dependent variable changes depending on the gender of the goat. *G_1i_* and *G_2i_* are genotype dummy variables (*i* = 3, the three genotypes were *G_1_*, *G_2_* and *G_3_*. *G_1i_* and *G_2i_* represent the two genotype dummy variables, with *G_3_* as the reference group), *S_1j_* is a dummy variable for sex, (*j* = male, female,when *j* = male, *S_1j_* = 1,when *j* = female, *S_1j_* = 0). *G_1i_S_1j_* and *G_2i_S_1j_* are the interaction terms for genotype and sex, which were included in the model to capture the combined effects of genotype and gender on the dependent variable. These terms allow us to explore how different genotypes of the *HMGCS1* gene interact with the sex of the Guizhou white goats to influence the dependent variables. For instance, it could be that a particular combination of genotype and sex leads to a more pronounced effect on slaughter characteristics or meat quality. By accounting for these interactions, we can better understand the underlying mechanisms and avoid oversimplifying the relationship. This not only helps in accurately predicting the outcomes but also provides more in-depth insights into the biological processes at play. Moreover, it enables us to make more refined interpretations of the results and potentially identify gender-specific genetic effects that might have been overlooked otherwise. The *e_ijk_* represents the random error, which accounts for the unexplained variability in the data. [38]. For multiple comparisons of different genotypes, we employed the Bonferroni correction. This correction was used to control the family-wise error rate, ensuring that the probability of making at least one Type I error (rejecting a true null hypothesis) across all comparisons was maintained at an acceptable level [26].

## RESULTS

### Analysis of slaughter characteristics, meat quality, and organ coefficients

Male and female goats showed differences in overall slaughtering performance. Various parameters such as pre-slaughter live weight, carcass weight, slaughter rate, net meat weight, net meat percentage, carcass net meat rate, GR value, and tail weight differed significantly between male and female goats (p<0.01). Additionally, back fat thickness differed between male and female goats, being more pronounced in male goats compared to female goats (p<0.05).

There were some differences in meat quality characteristics between male and female goats. The dry matter and protein content of female goat meat were significantly different from those of male goats (p<0.05), with female goat meat having higher values. Moreover, the pH value at 45 min in female goat meat was significantly different from that of male goats (p<0.01).

Significant differences were observed in organ coefficients between male and female goats. The heart, liver, and hoof coefficients were significantly higher in male goats compared to females (p<0.01). Additionally, the head coefficient was significantly greater in male goats than in female goats (p<0.05). In contrast, the large intestine, lung, and stomach coefficients were lower in female goats (p<0.01). For the remaining organs, no significant difference was found in organ coefficients (Table 3).

### Polymorphism analysis of the *HMGCS1* gene

**Figure 1:**
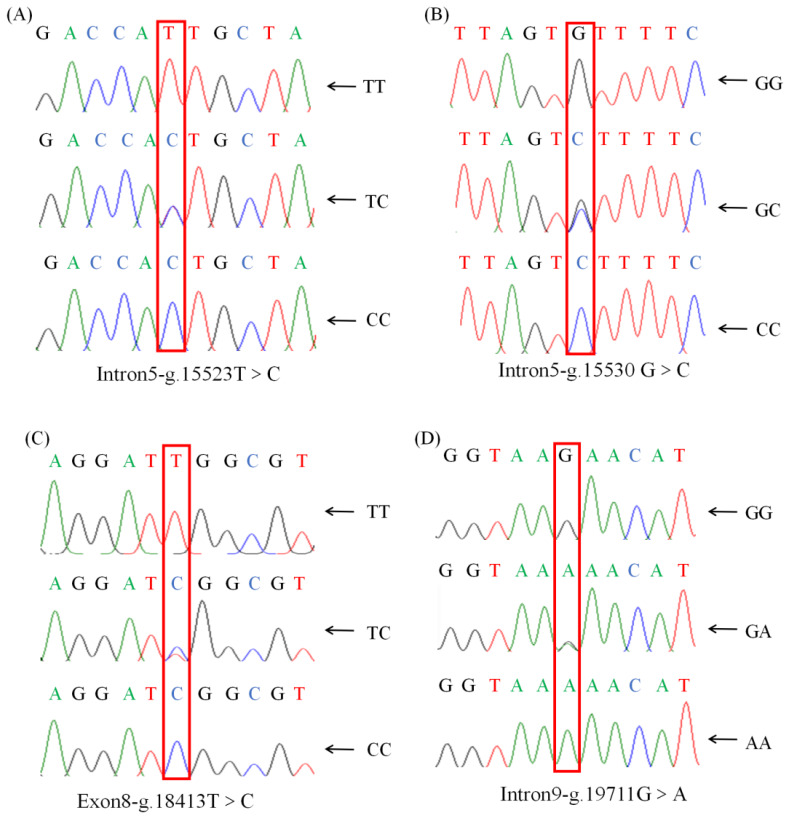
SNP locus of *HMGCS1* in Guizhou white goat. (A) g.15523 T>C. (B) g.15530 G>C. (C) g.18413 T>C. (D) g.19711 G>A. SNP, single nucleotide polymorphism.

**Figure 2:**
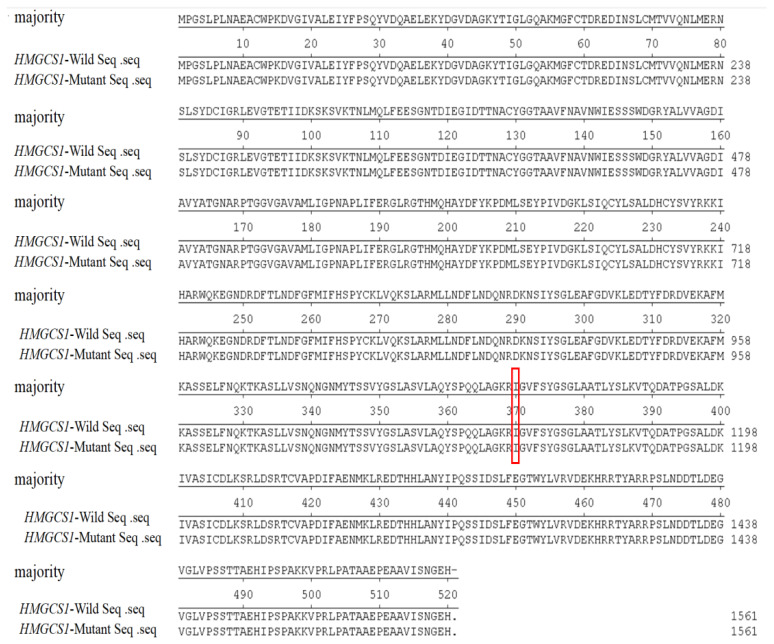
Alignment of wild-type and mutant *HMGCS1* amino acid sequences. Red box shows the synonymous SNP. SNP, single nucleotide polymorphism.

Sequence alignment of the *HMGCS1* gene in Guizhou white goats (accession number: NC_030827.1) led to the identification of four SNPs. In this study, the four SNPs identified in the *HMGCS1* gene of Guizhou white goats were labelled as g.15523T>C, g.15530G>C, g.18413T>C, and g.19711G>A. All of these SNPs exhibited two alleles and three genotypes (Figure 1). At the g.18413T>C locus, when comparing the wild and mutant-type, the base T was mutated to C. This alteration changed the codon from -ATT- to -ATC-. Interestingly, both codons encode the same amino acid, isoleucine (I), thus resulting in a synonymous SNP (Figure 2). Synonymous SNPs, although they do not change the amino acid sequence, can still have functional implications as they may affect mRNA stability, translation efficiency, or protein folding. The remaining SNPs (g.15523T>C, g.15530G>C, and g.19711G>A) were intronic mutations. Intronic SNPs are located within the non-coding introns and can potentially influence gene expression through mechanisms such as affecting splicing or interacting with regulatory elements. Population genetic analyses were conducted on these SNPs. The results indicated that the homozygosity at each SNP locus exceeded heterozygosity. Additionally, the PIC of these SNPs, which measures the amount of polymorphism in a population, ranged from 0.25<PIC<0.50 for g.15523T>C, g.15530G>C, g.18413T>C, and g.19711G>A, suggesting a moderate level of polymorphism. Chi-squared tests were performed to assess whether the observed genotype frequencies deviated from the expected frequencies under Hardy-Weinberg equilibrium. The results confirmed that all four SNP loci were in Hardy-Weinberg equilibrium (*p*>0.05) (Table 4).

### Linkage disequilibrium and haplotype analysis of the *HMGCS1* gene

**Figure 3:**
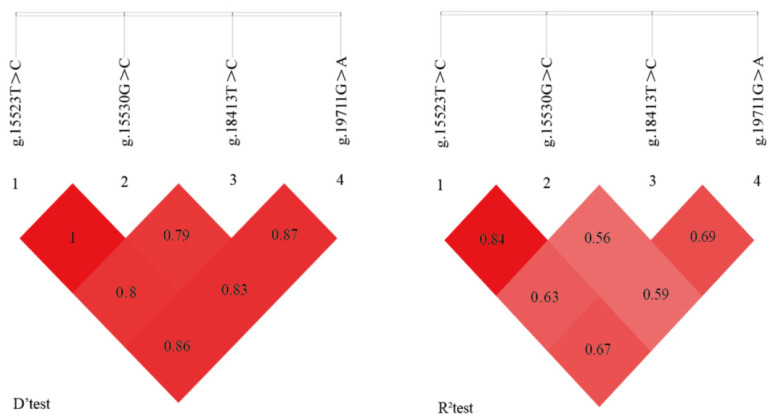
Analysis of linkage disequilibrium. r^2^ represents the correlation between a pair of loci, and D′ denotes the difference between the observed and the expected frequency of a given haplotype.

LD analysis of the *HMGCS1* SNPs exhibited D′ values ranging from 0.79 to 1.00 and r^2^ values from 0.54 to 0.85 (Figure 3) [39]. A strong chain imbalance was observed between SNPs g.15523 T>C and g.15530 G>C, with an r^2^ of 0.85 (Table 5). Haplotype analysis identified two dominant haplotypes in the Guizhou white goat population, namely, -CCCA-, -TGTG-, with frequencies of 57.59% and 42.41%, respectively. From haplotype combinations, three diplotypes were identified with frequencies above 5%. The most common diplotype, Hap1/2 (-TC-GC-TC-GA-), occurred in 56.56% of the population. Diplotypes Hap1/1 (-CC-CC-CC-AA-) and Hap2/2 (-TT-GG-TT-GG-) were present in 33.60% and 9.84%, respectively. Haplotypes and diplotypes with frequencies below 5% were not statistically significant and hence excluded from the statistical analysis (Table 6).

### Association between polymorphisms in the *HMGCS1* gene and slaughter characteristics, meat quality, and organ coefficients

In this study, sSNP genotypes were used as a reference for wild-type homozygous genotypes at mutant loci g.15523T> C(TT), g.15530G>C(GG), g.18413T>C(TT) and g.19711G> A(GG). Wild-type homozygous genotypes are the most prevalent and natural state in the studied livestock population. They are the basic benchmark for comparison with other genotypes and help to better understand the effects of SNP mutations on the traits of interest. As for the diplotype reference, we chose the most common diplotype Hap1/2 (-TC-GC-TC-GA-), which is present in 56.56% of the population. This decision was based on its high frequency, suggesting that it represents a typical or baseline state in livestock populations. By using Hap1/2 as a reference diplotype, we can more efficiently analyse the differences and associations of other diplotypes (e.g. Hap1/1 [-CC-CC-CC-AA-] and Hap2/2 [-TT-GG-TT-GG-]) with traits studied in the field of livestock production. This approach allows a more comprehensive understanding of the role of different genotypes and diplotypes in the traits studied in the field of animal science.

The association analysis revealed significant correlations between diplotypes and slaughter characteristics, meat quality traits, and organ coefficients (Table 7). The GR value of Guizhou white goat was significantly lower in diplotypes Hap1/1 (-CC-CC-CC-AA-) and Hap1/2 (-TC-TC-TC-GA-) compared to Hap2/2 (-TT-GG-TT-GG-) (p<0.05). The loin eye muscle area and a* values were significantly higher in Hap1/1 than in Hap1/2 and Hap2/2 (p<0.05). Conversely, shear force and stomach coefficients were significantly larger in diplotype Hap1/2 compared to Hap1/1 and Hap2/2 (p<0.05). The pH value at 45 min, fat content, and drip loss rate were significantly higher in diplotypes Hap2/2 compared to Hap1/1 and Hap1/2 (p<0.05). No significant differences were observed in other slaughter characteristics, meat quality traits, and organ coefficients across the three diploids (p>0.05).

Detailed associations between SNPs in the *HMGCS1* gene and slaughter characteristics, meat quality traits, organ coefficients are shown in Tables 8–11. For instance, the tail weight of the TT genotype at g.18413T>C was significantly lower than that of the CC genotype in male goats (p<0.05). Similarly, the loin eye muscle area was significantly larger in the TT genotype compared to TC genotypes in female goats (p<0.05). For most slaughter characteristics, male goats within the same genotype were extremely significantly higher than female goats (p<0.01). Among meat quality traits in male goats, the CC genotype was associated with significantly higher meat color a* values compared to the TC genotype, whereas the TT genotype was associated with higher pH at 45 min compared to the CC genotype (p<0.05). In female goats, protein content was significantly higher in the TT genotype compared to TC genotype, and shear force was highest in the TT genotype compared to TC and CC genotypes (p<0.05). Overall, male goats exhibited highly significantly lower dry matter and protein content but significantly higher cooked meat rate and shear force compared to female goats (p<0.01). Differences in organ coefficients, such as the liver, spleen, kidney, and hoof, were significant across different genotypes in male goats (p<0.05). Significant sex-based differences in organ coefficients were also observed for the same genotype (p<0.01) (Table 8). Further associations of SNPs g.15523T>C, g.15530G>C, and g.19711G>A with slaughter characteristics, meat quality traits, and organ coefficients in Guizhou white goats are shown in Tables 9–11.

## DISCUSSION

### Analysis of synonymous and intronic mutations in the *HMGCS1* gene

This study identified both synonymous and intronic SNPs in the *HMGCS1* gene. While synonymous SNPs did not alter the amino acid sequence in the *HMGCS1* gene, they can significantly influence meat quality traits. Similarly, intronic SNPs, despite not directly affecting protein coding, may impact growth performance. These findings align with prior research on other livestock species. For instance, synonymous SNPs in *SIRT3* and *POU1F1* have been significantly associated with goat body weight [40,41], while two synonymous SNPs in *SIRT2* were linked to weight traits in Nanyang cattle [42].

Several studies have consistently shown that synonymous SNPs can influence the stability and structure of mRNA, as well as protein folding, thereby affecting gene function and phenotypic traits [43]. Synonymous mutations, despite not altering the amino acid sequence, can introduce codon bias, which may alter the translation mechanism of the cell. This can potentially regulate gene expression, affect protein translation efficiency, and influence protein folding accuracy [44–46]. These changes in translation efficiency may lead to variations in protein expression levels and, consequently, phenotypic differences. For instance, an intronic SNP in the human *GRB10* gene has been associated with newborn birth weight [47]. Research suggests that certain SNPs in intronic regions can destabilize RNA and disrupt functional protein expression. They may also affect alternative splicing or interact with genetic elements such as enhancers, thereby regulating gene transcription or translation [48–50]. Therefore, these observations highlight the complex molecular mechanisms underlying the phenotypic impact of SNPs. Further research is needed to unravel these mechanisms, potentially enabling targeted breeding strategies to enhance livestock traits.

### Sex-based genetic differences in Guizhou white goats

This study revealed significant differences in pre-slaughter live weight and carcass weight between male and female goats of similar age (12-months-old) under identical feeding conditions. These findings are consistent with previous studies which have shown that live and carcass weights are influenced by both age and gender [51]. Moreover, significant differences in physiological characteristics and varying hormone levels between male and female goats have been identified. In particular, high estrogen levels are found in female goats, while testosterone dominates in male goats, which may exert an important regulatory role in gene expression [52]. Estrogen may affect gene expression related to fat deposition and muscle development, thus contributing to the differences in meat quality traits compared with male goats [53], which may explain the higher fat content and potentially better tenderness in female goat meat compared to male goat meat. Conversely, testosterone in male goats promotes muscle growth and metabolism, leading to higher lean meat percentages and distinctive muscle fiber characteristics [54].

Based on the data of the present study, it is evident that genotype performance varies between male and female goats, albeit the performance of specific genotypes in male and female goats showed a certain inconsistency. For instance, while no significant differences were found in shear force between male and female goats, the TC genotype showed significantly higher shear force in males compared to female goats (Tables 3, 8). This may be attributed to a higher lean meat percentage in male goats, whereas tenderness is more in female goats. Additionally, differences in certain meat quality traits such as dry matter and protein content between male and female goats may also reflect genotype-specific effects. For instance, certain genotypes (e.g., AA and CC) may play different roles in males and females (Tables 9, 11), influencing these traits differently.

Taken together, understanding sex-based genetic effects provides valuable insights into the relationship between genetics and meat quality traits. Such knowledge is important for advancing precision breeding and genetic improvement efforts in animal husbandry.

### Slaughter characteristics of Guizhou white goats

Slaughter characteristics and meat quality serve as crucial indicators for assessing breed resources and guiding breed selection and identification. For instance, indicators such as slaughter rate and net meat weight as slaughter characteristics can reflect the development phase of livestock [55]. Slaughter yield is influenced by a variety of factors, such as breed, age, gender, and seasonal factors, among which breed is the most influential [56]. Considering the carcass weight of male Guizhou white goats, it can be stated that it was significantly higher compared to Spanish goats [57], lower compared to Boer×Spanish goats [58], lower compared to Boer×Angora goats, but higher compared to purebred Angora goats [59]. Moreover, back fat thickness of Guizhou white goat was significantly lower compared to Boer×Guizhou white goats [60]. Thus, slaughter performance in Guizhou white goats is moderate and slightly inferior compared to that of crossbreed goats, suggesting a potential for selecting improved slaughter performance characteristics.

### Meat quality of Guizhou white goats

Meat quality traits such as dry matter, crude protein, and crude fat significantly influence the nutritional value of goat met [61,62]. This study highlighted key disparities in these traits between male and female Guizhou white goats. Males exhibited lower dry matter and protein content but higher pH value at 45 min postmortem compared to females (p<0.05). Intramuscular fat content and fatty acid composition are crucial factors of the quality of mutton, affecting tenderness, flavor, and nutritional value [63,64]. Compared to other breeds, Guizhou white goats showed protein contents similar to Korean Black goats but lower fat content, lighter meat color (L* and a* values), and higher pH [65]. Similar differences were noted against Boer goats [66]. These findings suggest that crossbreeding Guizhou white goats with breeds of higher meat quality could enhance their overall meat quality traits.

It has been shown that the expression of *HMGCS1* was positively associated with cholesterol content in goose liver, being considered a key gene in cholesterol synthesis [67]. Thus, we hypothesized that the *HMGCS1* gene could be related to intramuscular fat deposition in Guizhou white goats. However, the analysis of the association between the *HMGCS1* gene and meat quality traits in Guizhou white goats revealed that SNPs g.18413T>C, g.15523T>C, g.15530G>C, and g.19711G>A were significantly associated with meat color (a* and b* values), protein content, fat content, dry matter and shear force (p<0.05). No significant differences were found for other meat quality traits. In particular, the intronic SNPs g.15523T>C and g.15530G>C in the *HMGCS1* gene were significantly associated with intramuscular fat content (p<0.05). These two loci showed a strong LD state (D′ = 1, r^2^ = 0.84), which suggests a potential synergistic effect on phenotypic traits [39]. It has been suggested that the higher the degree of LD between two SNPs, the greater the likelihood that the two SNPs will be co-inherited in offspring in generational selection [68]. Thus, these novel LD-linked polymorphic loci could serve as critical targets for breeding Guizhou white goats with improved intramuscular fat content.

### Organ development in Guizhou white goats

Organ weight and organ indices provide valuable insights into animal genetics and physiology, serving as reference parameters for research and breeding [69]. However, these metrics are influenced by factors such as body weight, breed, sex, age, and environmental conditions [31]. In 12-month-old Guizhou white goats, organ weights and coefficients were approximately 50% lower than in adult goats [70], likely reflecting rapid early organ development. This fast growth phase may result in lower muscle and fat yields in 12-month-old goats, ultimately impacting meat production indicators [69].

The present study identified significant associations between the *HMGCS1* SNP loci and organ coefficients (p<0.05). Differences in organ coefficients among genotypes of Guizhou white goats underscore the complex relationship between genetics and physiology. Notably, most organ coefficients differed significantly between males and females of the same genotype, suggesting sex-based differences in growth rates and developmental patterns. These variations may affect organ growth rate and development, resulting in distinct organ coefficients between the sexes. Understanding the underlying mechanisms and functional consequences of these differences could inform breeding programs and disease management strategies, ultimately enhancing goat health and productivity.

## CONCLUSION

The present study constitutes the first investigation of specific *HMGCS1* SNPs and their combined effects on slaughter performance, meat quality traits, and organ coefficients in Guizhou white goats. The synonymous mutation at the g.18413T>C locus was significantly associated with meat color a* and b* values, dry matter, protein content, and shear force. Moreover, the intronic SNPs g.15523T>C and g.15530G> C in the *HMGCS1* gene were found to have strong LD and significantly associated with intramuscular fat content (p<0.05). Hence, these findings suggest that *HMGCS1* could serve as a valuable molecular marker for improving intramuscular fat content and slaughter performance in Guizhou white goats.

## Figures and Tables

**Table 1:** Composition and nutritional level of concentrate supplements given to Guizhou white goats

Composition	Content
Diet composition (%)	100.0
Corn	56.3
Soybean meal (CP 42%)	22.2
Rice bran	14.6
Calcium hydrogen phosphate	1.6
Mineral meal	1.0
Salt	0.3
Additive premix^1)^	4.0
Nutrient composition^2)^	
Digestible energy (MJ/kg)	13.85
Crude protein (%)	17.14
Calcium (%)	0.72
Total phosphorus (%)	0.51

1) Each kilogram of premix contains: Vitamin A 38,000 IU, Vitamin D 34,000 IU, Vitamin E 800 IU, Iron 350 mg, Zinc 1,000 mg, Manganese 700 mg, Copper 180 mg.

2) Digestible energy is a calculated value, the rest are measured values.

**Table 2:** Primers used to amplify the *HMGCS1* gene of Guizhou white goats^1)^

Primer	Primer sequences 5′→3′	Product size (bp)	Annealing temperature (°C)	Amplification region
P1	F: GTTCTCATCTCCTCTCGCTGAGCTT	572	63	Promoter, exon 1, intron 1
	R:AAACCTTAGCCCTGACCCGACA			
P2	F: GACCTGAAACTACCACTGTAAGAGC	574	63	Intron 1, exon 2, intron 2
	R:GTGACTGTTTCTGACTGAGCCAT			
P3	F: GCTCATACTATCATTGCTACCTCC	928	63	Intron 2, exon 3, intron 3
	R: ATGAGATGGCTAGATGGCATCACT			
P4	F: GCTCATACTATCATTGCTACCTCC	818	63	Intron 3, exon 4, intron 4
	R: ATGAGATGGCTAGATGGCATCACT			
P5	F: GTATATCAAGAGGCTTAGGCCTGTC	516	63	Intron 4, exon 5, intron 5
	R: CTGTGGGAAACCTATGCTATCTGG			
P6	F: GTGCTTGAGATACTGAGAAGGACAG	1,181	63	Intron 5, exon 6, intron 6
	R: GAAAACGGAAGGAGTATGAGAGC			
P7	F: CCTGTGTCCCTTCTCCATCTGATAA	689	63	Intron 6, exon 7, intron 7
	R: AGAGGCTAGAGAACCTCCAAGGAAA			
P8	F: TCACACTAGCTTTCCCTGTTGTTC	469	63	Intron 7, exon 8, intron 8
	R: CTTAAGCGTTCACTTGAGAAGCCT			
P9/10	F: TTCCTCAGTTGTCTACTAGCCAAGG	925	63	Intron 8, exon 9, intron 9, exon 10, intron 10
	R: CACCTCTCACCAAGATACAAACCAC			
P11	F: GTATGGTAGATAGATCTCCGTGGCC	576	63	Intron 10, exon 11
	R:GTAAGCATCGGCTAGACCACAACA			

1) The *HMGCS1* gene has 11 exons, and one exon mutation and three intron mutations were found in primer P8, P5, P9/10. The primer P9/10 contains the sequences of Exon - 9 and Exon - 10.

**Table 3:** The slaughter characteristics, meat quality traits and organ coefficient of Guizhou white goats

Indexes	Male goat	Female goat
Slaughter characteristics		
Live weight (kg)	37.13±0.55^**^	29.86±0.58
Carcass weight (kg)	16.37±0.25^**^	12.23±0.26
Dressing percentage (%)	44.12±0.43^**^	41.07±0.45
Net meat weight (kg)	12.71±0.24^**^	8.98±0.25
Net meat percentage (%)	34.16±0.46^**^	30.12±0.49
Carcass net meat rate (%)	77.38±0.78^**^	73.28±0.82
Loin eye muscle area (cm^2^)	10.15±0.28	9.90±0.29
GR value (mm)	7.29±0.20^**^	6.39±0.21
Back fat thickness (cm)	0.71±0.02^*^	0.63±0.02
Tail weight (g)	29.07±0.43^**^	22.41±0.45
Meat quality		
Meat color L	28.82±0.24	28.74±0.25
Meat color A	9.23±0.08	9.16±0.08
Meat color B	7.09±0.08	7.15±0.08
pH value (45min)	7.04±0.05^*^	6.87±0.05
Dry matter (%)	25.20±0.27^*^	26.32±0.28
Protein content (%)	21.33±0.22^*^	22.09±0.23
Fat content (%)	6.31±0.16	6.43±0.17
Drip loss rate (%)	1.52±0.08	1.53±0.09
Cooked meat rate (%)	62.39±0.62	60.68±0.65
Shear force (N)	90.67±1.84	86.50±1.94
Organ coefficients (%)		
Heart coefficients	0.56±0.02^**^	0.47±0.02
Liver coefficients	1.89±0.03^**^	1.58±0.03
Spleen coefficients	0.17±0.01	0.18±0.01
Lung coefficients	1.30±0.02^**^	1.41±0.02
Kidney coefficients	0.36±0.01	0.37±0.01
Small intestine coefficients	1.63±0.03	1.54±0.03
Large intestine coefficients	1.97±0.06^**^	2.39±0.06
Stomach coefficients	4.19±0.06^**^	4.50±0.07
Head coefficients	6.94±0.08^*^	6.63±0.09
Hoof coefficients	2.47±0.05^**^	2.03±0.05

* p<0.05,

** p<0.01,

no significant p>0.05.

**Table 4:** Analysis of Hardy Weinberg law of equilibrium of SNPs within the *HMGCS1* gene of Guizhou white goats

SNP locus	Genotype frequency			Gene frequency		χ^2^	p	*Ho*	*He*	*Ne*	*PIC*
g. 18413T>C	TT(21) 0.14	TC(80) 0.52	CC(52) 0.34	T 0.40	C 0.60	1.25	0.53	0.52	0.48	1.92	0.36
g. 15523T>C	TT(22) 0.15	TC(80) 0.52	CC(51) 0.33	T 0.41	C 0.59	1.10	0.58	0.52	0.48	1.93	0.37
g. 15530G>C	GG(22) 0.14	GC(83) 0.54	CC(48) 0.31	G 0.42	C 0.58	2.10	0.35	0.51	0.49	1.94	0.37
g. 19711G>A	GG(18) 0.12	GA(80) 0.52	AA(55) 0.36	G 0.38	A 0.62	1.88	0.39	0.53	0.47	1.89	0.36

A *PIC* value<0.25 indicates low polymorphism; 0.25<*PIC*<0.50 indicates moderate polymorphism; and *PIC* > 0.5 indicates high polymorphism.

χ^2^_0.05_ = 5.99; χ^2^_0.01_ = 9.21; χ^2^<5.99 with p>0.05 indicates that the population is in Hardy-Weinberg equilibrium.

SNP, single nucleotide polymorphism; *Ho*, homozygosity; *He*, for heterozygosity; *Ne*, effective number of alleles; *PIC*, polymorphic information content.

**Table 5:** Linkage disequilibrium coefficient between SNPs of *HMGCS1*

D′\r^2^	g.15523 T>C	g.15530 G>C	g.18413 T>C	g.19711 G>A
g.15523 T>C	-	0.85	0.61	0.66
g.15530 G>C	1.00	-	0.54	0.58
g.18413 T>C	0.79	0.79	-	0.69
g.19711 G>A	0.86	0.83	0.863	-

SNP, single nucleotide polymorphism.

**Table 6:** *HMGCS1* gene haplotype analysis and frequency

Haplotype and diplotype	g.15523 T>C	g.15530 G>C	g.18413 T>C	g.19711 G>A	Frequency (%)
Hap1	C	C	C	A	57.59
Hap2	T	G	T	G	42.41
Hap1/1	CC	CC	CC	AA	33.60
Hap1/2	TC	GC	TC	GA	56.56
Hap2/2	TT	GG	TT	GG	9.84

**Table 7:** Association analysis of *HMGCS1* gene diplotype with slaughter traits, meat quality traits and organ coefficient

Indexes	Diplotype		
Slaughter characteristics	Hap1/1	Hap1/2	Hap2/2
Live weight (kg)	34.24±6.6	32.83±4.74	30.94±5.85
Carcass weight (kg)	14.63±3.06	13.94±2.53	13.31±3.31
Dressing percentage (%)	42.74±3.56	42.42±4.03	42.71±3.73
Net meat weight (kg)	11.25±2.80	10.43±2.31	9.85±2.55
Net meat percentage (%)	32.77±4.18	31.63±4.31	31.52±3.02
Carcass net meat rate (%)	76.29±6.42	74.37±6.42	74.23±3.94
Loin eye muscle area (cm^2^)	10.53±2.36^a^	9.49±1.92^b^	10.15±3.24^ab^
GR value (mm)	7.08±1.71	6.5±1.61	6.39±2.32
Back fat thickness (cm)	0.66±0.17	0.66±0.18	0.65±0.15
Tail weight(g)	25.79±5.20	25.25±4.88	23.51±2.67
Meat quality			
Meat color L	28.94±2.24	28.86±1.60	28.33±2.76
Meat color A	9.26±0.76^a^	8.98±0.55^b^	9.25±0.69^ab^
Meat color B	7.00±0.59	7.21±0.59	7.38±0.49
pH value (45min)	6.84±0.43^a^	6.93±0.37^ab^	7.12±0.53^b^
Dry matter (%)	26.18±2.34	25.45±2.33	26.47±1.63
Protein content (%)	21.55±2.09	21.53±1.72	22.53±1.25
Fat content (%)	6.06±1.50^ab^	6.05±1.30^a^	6.88±0.86^b^
Drip loss rate (%)	1.41±0.56^a^	1.47±0.60^ab^	1.87±0.67^b^
Cooked meat rate (%)	60.42±8.42	61.27±2.71	62.5±3.35
Shear force (N)	83.45±15.59^a^	89.31±11.72^b^	88.98±21.45^ab^
Organ coefficients (%)			
Heart coefficients	0.51±0.13	0.50±0.11	0.49±0.06
Liver coefficients	1.72±0.24	1.78±0.34	1.61±0.26
Spleen coefficients	0.18±0.03	0.18±0.03	0.18±0.02
Lung coefficients	1.36±0.15	1.36±0.14	1.40±0.13
Kidney coefficients	0.38±0.12	0.38±0.12	0.36±0.06
Small intestine coefficients	1.57±0.17	1.56±0.24	1.62±0.39
Large intestine coefficients	2.22±0.44	2.26±0.49	2.3±0.47
Stomach coefficients	4.23±0.45^a^	4.43±0.48^b^	4.42±0.57^ab^
Head coefficients	6.70±0.67	6.74±0.63	6.58±0.55
Hoof coefficients	2.19±0.28	2.17±0.3	2.10±0.26

Diplotypes with <5.0% frequency were not considered.

Different capital letters and different lowercase letters indicate, respectively, highly significant difference (p<0.01) and significant difference (p<0.05). If the characters were identical, no significant difference was indicated (p>0.05).

**Table 8:** Comparison of slaughtering traits, meat quality traits and organ coefficients among different genotypes of Guizhou white goats at g.18413T>C locus^1)^

Indexes	Male goat			Female goat		
	
	TT	TC	CC	TT	TC	CC
Slaughter characteristics						
Live weight (kg)	38.06±1.27^**^	35.97±0.68^**^	37.35±0.78^**^	28.73±1.33	30.59±0.65	30.25±0.88
Carcass weight (kg)	16.72±0.58^**^	16.07±0.31^**^	16.31±0.36^**^	11.85±0.61	12.46±0.30	12.39±0.40
Dressing percentage (%)	43.87±1.00	44.83±0.54^**^	43.67±0.62^**^	41.22±1.05	40.75±0.51	41.25±0.69
Net meat weight (kg)	12.94±0.55^**^	12.34±0.29^**^	12.85±0.34^**^	8.73±0.57	9.05±0.28	9.15±0.38
Net meat percentage (%)	33.82±1.08	34.38±0.58^**^	34.29±0.66^**^	30.30±1.13	29.58±0.55	30.48±0.75
Carcass net meat rate (%)	77.13±1.82	76.47±0.98^**^	78.54±1.12^**^	73.90±1.91	72.64±0.93	73.29±1.26
Loin eye muscle area (cm^2^)	9.85±0.64	9.91±0.34	10.67±0.39	10.56±0.67^a^	9.07±0.33^b^	10.07±0.44^ab^
GR value (mm)	7.09±0.47	7.24±0.25^**^	7.53±0.29	6.46±0.50	6.08±0.24	6.63±0.33
Back fat thickness (cm)	0.77±0.05	0.69±0.03	0.68±0.03	0.63±0.05	0.65±0.03	0.62±0.04
Tail weight (g)	27.53±1.00^a**^	29.38±0.54^ab**^	30.30±0.62^b**^	23.12±1.05	22.27±0.51	21.84±0.69
Meat quality						
Meat color L	29.13±0.5	28.67±0.30	28.68±0.35	27.86±0.59	29.00±0.29	29.35±0.39
Meat color A	9.37±0.19^ab^	8.99±0.10^a^	9.34±0.12^b^	9.28±0.20	9.03±0.10	9.19±0.13
Meat color B	7.02±0.18	7.23±0.10	7.02±0.11	7.22±0.19	7.21±0.09	7.03±0.13
pH value (45 min)	7.21±0.11^a^	7.02±0.06^ab^	6.89±0.07^b^	6.91±0.12	6.88±0.06	6.82±0.08
Dry matter (%)	25.68±0.62	25.02±0.33	24.89±0.38	26.19±0.65	25.87±0.32	26.89±0.43^**^
Protein content (%)	20.95±0.50	21.65±0.27	21.38±0.31	22.75±0.53^a*^	21.48±0.26^b^	22.06±0.35^ab^
Fat content (%)	6.62±0.38	6.11±0.20	6.19±0.23	6.88±0.40	6.12±0.19	6.29±0.26
Drip loss rate (%)	1.72±0.19	1.46±0.10	1.40±0.16	1.53±0.20	1.49±0.10	1.57±0.13
Cooked meat rate (%)	63.53±1.44	61.57±0.78	62.06±0.89^*^	61.41±1.51	61.34±0.74	59.29±1.00
Shear force (N)	86.99±4.28^a^	96.69±2.30^b**^	88.34±2.64^a^	89.75±4.49^a^	87.41±2.19^b^	82.34±2.96^b^
Organ coefficients (%)						
Heart	0.60±0.04^*^	0.54±0.02^**^	0.52±0.02	0.48±0.04	0.46±0.02	0.48±0.02
Liver	1.80±0.07^A*^	2.03±0.04^B**^	1.85±0.05^A**^	1.53±0.08	1.60±0.04	1.61±0.05
Spleen	0.16±0.01^a^	0.19±0.01^b^	0.17±0.01^ab^	0.19±0.01^*^	0.18±0.01	0.18±0.01
Lung	1.28±0.04^**^	1.33±0.02	1.30±0.03	1.44±0.05	1.38±0.02	1.40±0.03
Kidney	0.33±0.03^a^	0.40±0.02^b*^	0.35±0.02^a^	0.38±0.03	0.34±0.02	0.39±0.02
Small intestine	1.59±0.07	1.67±0.04^**^	1.63±0.04	1.58±0.07	1.51±0.04	1.54±0.05
Large intestine	1.94±0.13	2.03±0.07	1.95±0.08	2.42±0.13^**^	2.41±0.07^**^	2.34±0.09^**^
Stomach	4.18±0.15	4.28±0.08	4.11±0.09	4.56±0.16	4.56±0.08^*^	4.37±0.10
Head	7.06±0.19	6.92±0.10^*^	6.83±0.12	6.58±0.20	6.61±0.10	6.70±0.13
Hoof	2.75±0.12^A**^	2.36±0.06^B**^	2.28±0.07^B*^	2.03±0.13	2.01±0.06	2.06±0.08

1) Letter, intra-group comparison of males or females; *, **, comparison between groups within the same genotype.

A,B Statistically significant different at significance level of p<0.01.

a,b Statistically significant differences at significance level of p<0.05.

* p<0.05 (significant),

** p<0.01 (extremely significant).

**Table 9:** Comparison of slaughtering traits, meat quality traits and organ coefficients among different genotypes of Guizhou white goats at g.15523T>C locus

Indexes	Male goat			Female goat		
	
	TT	TC	CC	TT	TC	CC
Slaughter characteristics						
Live weight (kg)	38.52±1.26^a**^	35.64±0.66^b**^	37.77±0.80^a**^	28.49±1.26	30.57±0.66	30.49±0.85
Carcass weight (kg)	17.00±0.57^a**^	15.74±0.30^b**^	16.70±0.36^a**^	11.70±0.57	12.46±0.30	12.48±0.39
Dressing percentage (%)	44.14±1.01^*^	44.28±0.53^**^	44.29±0.64^**^	41.03±1.01	40.80±0.53	41.21±0.68
Net meat weight (kg)	13.01±0.54^ab**^	12.10±0.28^a**^	13.22±0.34^b**^	8.58±0.54	9.02±0.28	9.27±0.36
Net meat percentage (%)	33.71±1.07^*^	33.96±0.56^**^	34.96±0.68^**^	29.98±1.07	29.52±0.56	30.66±0.73
Carcass net meat rate (%)	76.35±1.81	76.51±0.95^**^	78.95±1.16^**^	73.42±1.81	72.39±0.95	73.85±1.23
Loin eye muscle area (cm^2^)	9.31±0.64	10.05±0.33	10.75±0.41	10.88±0.64^a^	9.12±0.33^b^	9.74±0.43^ab^
GR value (mm)	7.65±0.47	7.07±0.25^**^	7.58±0.30^*^	6.41±0.47	6.09±0.25	6.62±0.32
Back fat thickness (cm)	0.71±0.05	0.69±0.03	0.71±0.03	0.64±0.05	0.64±0.03	0.63±0.04
Tail weight (g)	27.85±1.01^**^	29.69±0.53^**^	29.78±0.65^**^	22.73±1.01	22.36±0.53	21.85±0.69
Meat quality						
Meat color L	28.97±0.56	28.77±0.30	28.59±0.36	28.03±0.56	29.10±0.30	29.15±0.38
Meat color A	9.23±0.19	9.07±0.10	9.31±0.12	9.26±0.19	9.03±0.10	9.18±0.13
Meat color B	7.39±0.18	7.14±0.09	6.98±0.12	7.20±0.18	7.22±0.09	7.03±0.12
pH value (45 min)	7.16±0.12	7.02±0.06	6.91±0.07	6.88±0.12	6.86±0.06	6.87±0.08
Dry matter (%)	26.30±0.61^a^	24.71±0.32^b^	25.09±0.39^ab^	26.12±0.61^ab^	25.83±0.32^a*^	26.94±0.41^b**^
Protein content (%)	21.26±0.50	21.69±0.26^*^	21.17±0.32	22.68±0.50^a^	21.46±0.26^b^	22.04±0.34^ab^
Fat content (%)	5.90±0.38	6.33±0.20	6.16±0.24	6.95±0.38^a*^	6.06±0.20^b^	6.33±0.26^ab^
Drip loss rate (%)	1.65±0.19	1.49±0.10	1.38±0.12	1.67±0.19	1.49±0.10	1.50±0.13
Cooked meat rate (%)	62.01±1.45	61.86±0.76	62.29±0.93	61.45±1.45	61.09±0.76	59.76±0.98
Shear force (N)	95.80±4.30^ab^	94.77±2.25^a*^	86.98±2.74^b^	89.39±4.30	87.74±2.25	82.07±2.91
Organ coefficients (%)						
Heart	0.52±0.04	0.53±0.02^**^	0.57±0.02^**^	0.47±0.04	0.46±0.02	0.48±0.02
Liver	1.80±0.07^A*^	2.03±0.04^B**^	1.84±0.05^A**^	1.54±0.07	1.59±0.04	1.63±0.05
Spleen	0.16±0.01	0.18±0.01	0.17±0.01	0.19±0.01	0.18±0.01	0.19±0.01
Lung	1.21±0.04^Aa^	1.32±0.02^b^	1.35±0.03^B^	1.45±0.04^**^	1.38±0.02	1.40±0.03
Kidney	0.29±0.03^Aa^	0.40±0.02^B**^	0.36±0.02^b^	0.37±0.03	0.34±0.02	0.39±0.02
Small intestine	1.60±0.07	1.70±0.04^**^	1.59±0.04	1.58±0.07^ab^	1.47±0.04^a^	1.60±0.05^b^
Large intestine	1.81±0.13	2.00±0.07	2.03±0.08	2.37±0.13^**^	2.40±0.07^**^	2.38±0.09^**^
Stomach	3.96±0.15^a^	4.30±0.08^b^	4.15±0.10^ab^	4.53±0.15^**^	4.54±0.08^*^	4.43±0.10^*^
Head	6.86±0.19	6.94±0.10^*^	6.88±0.12	6.56±0.19	6.62±0.10	6.68±0.13
Hoof	2.27±0.12	2.38±0.06^**^	2.46±0.08^**^	1.99±0.12	2.02±0.06	2.07±0.08

1) Letter, intra-group comparison of males or females; *, **, comparison between groups within the same genotype.

A,B Statistically significant different at significance level of p<0.01.

a,b Statistically significant differences at significance level of p<0.05.

* p<0.05 (significant),

** p<0.01 (extremely significant).

**Table 10:** Comparison of slaughtering traits, meat quality traits and organ coefficients among different genotypes of Guizhou white goats at g.15530 G>C locus

Indexes	Male goat			Female goat		
	
	GG	GC	CC	GG	GC	CC
Slaughter characteristics						
Live weight (kg)	38.52±1.26^a**^	35.72±0.65^b**^	37.72±0.82^ab**^	28.49±1.26	30.59±0.64	30.44±0.89
Carcass weight (kg)	17.00±0.57^ab**^	15.76±0.30^a**^	16.71±0.37^b**^	11.70±0.57	12.46±0.30	12.49±0.40
Dressing percentage (%)	44.14±1.01^*^	44.23±0.52^**^	44.38±0.66^**^	41.03±1.01	40.75±0.52	41.35±0.71
Net meat weight (kg)	13.01±0.54^AB**^	12.1±0.28^A**^	13.26±0.35^B**^	8.58±0.54	9.05±0.27	9.25±0.38
Net meat percentage (%)	33.71±1.07^*^	33.88±0.55^**^	35.11±0.70^**^	29.98±1.07	29.58±0.55	30.65±0.76
Carcass net meat rate (%)	76.35±1.81	76.44±0.94^**^	79.15±1.18^**^	73.42±1.81	72.64±0.93	73.5±1.28
Loin eye muscle area (cm^2^)	9.31±0.63^a^	9.99±0.33^ab*^	10.87±0.41^b^	10.88±0.63^a^	9.07±0.32^b^	9.89±0.45^ab^
GR value (mm)	7.65±0.47	7.1±0.24^**^	7.55±0.31	6.41±0.47	6.08±0.24	6.67±0.33
Back fat thickness (cm)	0.71±0.05	0.68±0.03	0.72±0.03	0.64±0.05	0.65±0.03	0.62±0.04
Tail weight (g)	27.85±1.01^**^	29.63±0.53^**^	29.88±0.66^**^	22.73±1.01	22.27±0.52	21.98±0.72
Meat quality						
Meat color L	28.97±0.56	28.73±0.29	28.65±0.37	28.03±0.56	29.00±0.29	29.33±0.40
Meat color A	9.23±0.19	9.05±0.10	9.35±0.12	9.26±0.19	9.03±0.10	9.19±0.13
Meat color B	7.39±0.18^a^	7.15±0.09^ab^	6.96±0.12^b^	7.20±0.18	7.21±0.09	7.03±0.13
pH value (45 min)	7.16±0.12	7.02±0.06	6.9±0.07	6.88±0.12	6.88±0.06	6.84±0.08
Dry matter (%)	26.3±0.61^a^	24.67±0.31^b^	25.17±0.39^ab^	26.12±0.61^ab^	25.87±0.31^a**^	26.95±0.43^b**^
Protein content (%)	21.26±0.50	21.69±0.26	21.15±0.33	22.68±0.50^a*^	21.48±0.26^b^	22.06±0.35^ab^
Fat content (%)	5.90±0.38	6.36±0.20	6.11±0.25	6.95±0.38^a*^	6.12±0.19^b^	6.23±0.27^ab^
Drip loss rate (%)	1.65±0.19	1.49±0.10	1.37±0.12	1.67±0.19	1.49±0.10	1.5±0.13
Cooked meat rate (%)	62.01±1.45	61.98±0.75	62.12±0.94^*^	61.45±1.45	61.34±0.74	59.17±1.02
Shear force (N)	95.8±4.29^ab^	94.88±2.22^a*^	86.52±2.79^b^	89.39±4.29	87.41±2.20	82.18±3.04
Organ coefficients (%)						
Heart	0.52±0.04	0.53±0.02^**^	0.57±0.02^**^	0.47±0.04	0.46±0.02	0.48±0.03
Liver	1.80±0.07^A**^	2.03±0.04^B*^	1.84±0.05^A**^	1.54±0.07	1.60±0.04	1.61±0.05
Spleen	0.16±0.01	0.18±0.01	0.17±0.01	0.19±0.01	0.18±0.01	0.18±0.01
Lung	1.21±0.04^Aa^	1.31±0.02^b^	1.36±0.03^Bb^	1.45±0.04^**^	1.38±0.02^*^	1.40±0.03
Kidney	0.29±0.03^Aa^	0.4±0.02^Bb*^	0.36±0.02^b^	0.37±0.03	0.34±0.02	0.39±0.02
Small intestine	1.60±0.07	1.70±0.04^**^	1.59±0.05	1.58±0.07	1.51±0.04	1.54±0.05
Large intestine	1.81±0.13	2.00±0.07	2.04±0.08	2.37±0.13^**^	2.41±0.07^**^	2.35±0.09^*^
Stomach	3.96±0.15	4.29±0.08	4.17±0.10	4.53±0.15^**^	4.56±0.08^*^	4.38±0.11^**^
Head	6.86±0.19	6.95±0.10^*^	6.86±0.13	6.56±0.19	6.61±0.10	6.72±0.14
Hoof	2.27±0.12	2.37±0.06^**^	2.46±0.08^**^	1.99±0.12	2.01±0.06	2.08±0.09

1) Letter, intra-group comparison of males or females; *, **, comparison between groups within the same genotype.

A,B Statistically significant different at significance level of p<0.01.

a,b Statistically significant differences at significance level of p<0.05.

* p<0.05 (significant),

** p<0.01 (extremely significant).

**Table 11:** Comparison of slaughtering traits, meat quality traits and organ coefficients among different genotypes of Guizhou white goats at g.19711 G>A locus

Indexes	Male goat			Female goat		
	
	GG	GA	AA	GG	GA	AA
Slaughter characteristics						
Live weight (kg)	36.78±1.50^**^	36.44±0.70^**^	37.16±0.74^**^	28.19±1.34	30.4±0.65	30.85±0.90
Carcass weight (kg)	16.46±0.68^**^	16.15±0.32^**^	16.30±0.33^**^	11.62±0.61	12.40±0.29	12.61±0.41
Dressing percentage (%)	44.70±1.18^*^	44.48±0.55^**^	43.92±0.58^**^	41.16±1.06^a^	40.82±0.51^ab^	41.15±0.71^b^
Net meat weight (kg)	11.90±0.64^**^	12.69±0.30^**^	12.7±0.32^**^	8.10±0.57^a^	8.98±0.28^b^	9.33±0.39b
Net meat percentage (%)	32.14±1.25^a^	34.9±0.58^b**^	34.07±0.61^ab**^	30.38±1.12	29.55±0.54	30.54±0.75
Carcass net meat rate (%)	71.71±2.09^a^	78.29±0.97^b**^	77.63±1.03^b*^	74.20±1.87	72.45±0.90	73.55±1.26
Loin eye muscle area (cm^2^)	9.28±0.75	10.26±0.35^*^	10.32±0.37	10.94±0.67	9.03±0.32	10.04±0.45
GR value (mm)	6.96±0.55	7.18±0.26^**^	7.58±0.27^*^	6.54±0.49	6.06±0.24	6.67±0.33
Back fat thickness (cm)	0.7±0.06	0.71±0.03	0.69±0.03	0.65±0.06	0.65±0.03	0.61±0.04
Tail weight (g)	26.90±1.18^a*^	29.88±0.55^b**^	29.61±0.58^b**^	23.13±1.05	22.19±0.51	21.97±0.71
Meat quality						
Meat color L	28.81±0.66	28.75±0.31	28.7±0.33	28.07±0.59	28.98±0.29	29.3±0.40
Meat color A	9.28±0.22	9.06±0.10	9.28±0.11	9.27±0.20	9.02±0.10	9.20±0.13
Meat color B	7.45±0.21	7.14±0.10	7.02±0.10	7.20±0.19	7.20±0.09	7.05±0.13
pH value (45 min)	7.21±0.14	7.00±0.06	6.95±0.07	6.91±0.12	6.89±0.06	6.80±0.08
Dry matter (%)	25.54±0.72	25.07±0.34	24.94±0.36	26.14±0.65^ab^	25.86±0.31^a^	26.98±0.44^b**^
Protein content (%)	21.83±0.59	21.55±0.27	21.24±0.29	22.81±0.53^a^	21.49±0.25^b^	22.03±0.35^ab^
Fat content (%)	6.09±0.44	6.39±0.21	6.05±0.22	6.99±0.40	6.16±0.19	6.18±0.27
Drip loss rate (%)	1.88±0.22	1.43±0.10	1.43±0.11	1.74±0.19	1.52±0.09	1.40±0.13
Cooked meat rate (%)	62.89±1.69	61.99±0.79	61.87±0.83^*^	61.29±1.52	61.4±0.73	59.13±1.02
Shear force (N)	91.71±4.64	91.37±2.16	88.08±2.28	88.77±4.15	87.4±2.00	82.57±2.80
Organ coefficients (%)						
Heart	0.52±0.04	0.54±0.02^*^	0.55±0.02^*^	0.47±0.04	0.46±0.02	0.48±0.03
Liver	2.01±0.09^**^	1.96±0.04^**^	1.88±0.04^**^	1.54±0.08	1.60±0.04	1.60±0.05
Spleen	0.18±0.01	0.18±0.01	0.18±0.01	0.18±0.01	0.18±0.01	0.18±0.01
Lung	1.28±0.05	1.31±0.02	1.33±0.02	1.45±0.05^*^	1.39±0.02^*^	1.38±0.03
Kidney	0.32±0.04^a^	0.40±0.02^b*^	0.35±0.02^a^	0.37±0.03	0.34±0.02	0.39±0.02
Small intestine	1.63±0.08	1.66±0.04^*^	1.64±0.04	1.61±0.07	1.51±0.04	1.53±0.05
Large intestine	1.91±0.15	1.99±0.07	2.00±0.07	2.35±0.13^*^	2.41±0.06^**^	2.37±0.09^*^
Stomach	4.11±0.18	4.19±0.08	4.24±0.09	4.56±0.16	4.56±0.08^*^	4.36±0.11
Head	6.97±0.23	6.95±0.10^*^	6.84±0.11	6.51±0.20	6.62±0.10	6.71±0.14
Hoof	2.38±0.15^*^	2.37±0.07^**^	2.41±0.07^*^	1.99±0.13	2.01±0.06	2.08±0.09

1) Letter, intra-group comparison of males or females; *, **, comparison between groups within the same genotype.

A,B Statistically significant different at significance level of p<0.01.

a,b Statistically significant differences at significance level of p<0.05.

* p<0.05 (significant),

** p<0.01 (extremely significant).
